# Gastrointestinal Symptoms Associated With Unfavorable Prognosis of COVID-19 Patients: A Retrospective Study

**DOI:** 10.3389/fmed.2020.608259

**Published:** 2020-11-11

**Authors:** Rong Chen, Yan-li Yu, Wei Li, Ya Liu, Jing-xiao Lu, Fangyue Chen, Qin Zhou, Zhong-yuan Xia, Ling Gao, Qing-tao Meng, Daqing Ma

**Affiliations:** ^1^Department of Anesthesiology, Renmin Hospital of Wuhan University, Wuhan, China; ^2^Department of Anesthesiology, East Hospital, Renmin Hospital of Wuhan University, Wuhan, China; ^3^Department of Gastroenterology, Renmin Hospital of Wuhan University, Wuhan, China; ^4^Department of General Medicine, Peterborough City Hospital, North West Anglia NHS Foundation Trust, Peterborough, United Kingdom; ^5^Department of Endocrinology and Metabolism, Renmin Hospital of Wuhan University, Wuhan, China; ^6^Division Anaesthetics, Pain Medicine and Intensive Care, Department of Surgery and Cancer, Faculty of Medicine, Imperial College London, Chelsea and Westminster Hospital, London, United Kingdom

**Keywords:** gastrointestinal symptoms, COVID-19, prognosis, SARS-CoV-2 (COVID-19), ARDS (acute respiratory distress syndrome)

## Abstract

**Background and Aim:** The global pandemic of COVID-19 has posed an enormous threat to the economy and people's lives across various countries. Patients with COVID-19 most commonly present with respiratory symptoms. However, gastrointestinal (GI) symptoms can also occur. We aimed to study the relationship between GI symptoms and disease prognosis in patients with COVID-19.

**Methods:** In a single-center and retrospective cohort study, the outcomes in COVID-19 patients with or without GI symptoms were compared. The propensity score is a conditional probability of having a particular exposure (COVID-19 patients with GI symptoms vs. without GI symptoms) given a set of baseline measured covariates. Survival was estimated using the Kaplan-Meier method, and any differences in survival were evaluated with a stratified log-rank-test. To explore the GI symptoms associated with ARDS, non-invasive ventilator treatment, tracheal intubation, tracheotomy, and CRRT, univariable and multivariable COX regression models were used.

**Results:** Among 1,113 eligible patients, 359 patients with GI symptoms and 718 without GI symptoms had similar propensity scores and were included in the analyses. Patients with GI symptoms, as compared with those without GI symptoms, were associated with a similar risk of death, but with higher risks of ARDS, non-invasive mechanical ventilation in COVID-19 patients, respectively.

**Conclusions:** The presence of GI symptoms was associated with a high risk of ARDS, non-invasive mechanical ventilation and tracheal intubation in patients with COVID-19 but not mortality.

## Introduction

The global pandemic of COVID-19 has posed an enormous threat to the economy and people's lives across various countries (1, 2). The clinical spectrum of COVID-19 appears to be wide, ranging from asymptomatic infection, mild to critically-ill cases (3–6). Significant comorbidities such as type 2 diabetes mellitus (T2DM) and cardiovascular diseases (CVDs) were associated with developing severe and critical COVID-19 condition (7, 8). In severe cases, patients can rapidly develop acute respiratory distress syndrome (ARDS), septic shock, and multiple organ dysfunction syndromes (9). The most common symptoms of COVID-19 are fever, cough, fatigue, myalgia, and dyspnoea (10). Gastrointestinal (GI) symptoms were also observed in a significant proportion of patients (11–13), which were possibly due to the enrichment and infection of SARS-CoV-2 in the gastrointestinal tract.

Recent studies showed that angiotensin converting enzyme 2 (ACE2) plays a crucial role in the cellular infection with SARS-CoV-2 virus (14–16). Although ACE2 was found to be widely expressed across tissues, it was considered to be intestine-specific, and was enriched more than 4-fold in the epithelia of the intestinal tract compared with other tissues (17). SARS-CoV-2 disrupts ACE2 activity and infects the intestinal epithelium by inducing cytotoxicity (18), and then it is shed into feces, resulting in GI symptoms and/or positive SARS-CoV-2 viral load or RNA in stool (19). It is known that gastrointestinal problems in critically-ill patients were common and were associated with unfavorable outcomes (20). Trillions of diverse bacteria located in the intestinal tract and constituted the intestinal “microbiota” (21). Our previous studies found that bacteria and toxins enter into blood after intestinal mucosa injury caused by adverse stimulates, leading to damage of multiple remote organs (22). The impact of intestinal mucosa injured by SARS-CoV-2 infection and consequence on prognosis in patients with COVID-19 remains unknown. In this study, we investigated patients with GI symptoms, who were admitted to Renmin hospital of Wuhan University, Wuhan, China, associated with prognosis or outcome in patients with COVID-19.

## Materials and Methods

### Study Design and Participants

This single-center, retrospective cohort study included two cohorts of inpatients from East Campus of Renmin Hospital of Wuhan University. It was approved by the Institutional Review Board at Renmin hospital of Wuhan University (No. WDRY2020-K111, March 12, 2020) and have been performed in accordance with the ethical standards laid down in an appropriate version of the Declaration of Helsinki (as revised in Brazil 2013). Due to the urgency of this infectious disease, data analysis was performed anonymously and written informed consent was exempted. The East Campus of Renmin Hospital of Wuhan University is one of the major hospitals designated by the government to be responsible for patients with COVID-19 who are critically-ill, pregnant, or require surgery from January 25, 2020. This study included a total of 1,117 hospitalized patients with COVID-19 from January 25, 2020 to March 31, 2020. The diagnosis of COVID-19 according to the diagnostic criteria established by WHO and the New Coronavirus Pneumonia Prevention and Control Program (5th−7th edition) (23–25) issued by the National Health Commission of China. COVID-19 patients were diagnosed with clinical symptoms together with nasopharyngeal swabs tested positive for SARS-CoV-2 using real-time reverse transcription PCR (RT-PCR). All patients received chest radiography or chest CT scan on admission. Patients were divided into groups with gastrointestinal (GI) symptom or without GI symptom according to the presence or absence of GI symptoms.

### Data Collection

All information including epidemiological, demographic, clinical, laboratory, treatment, and outcome data were extracted from the medical record system of the Renmin Hospital of Wuhan University, and were collected and reviewed by three investigators using a standardized data collection form. All data were collected including age, sex, exposure history, comorbidities (hypertension, diabetes, coronary heart disease, cerebrovascular disease, chronic heart failure, liver dysfunction, chronic kidney disease, and chronic pulmonary disease), GI symptoms (abdominal pain, acid reflux, nausea or vomiting, abdominal distension, diarrhea, tenesmus, and belching), common symptoms (fever, cough, chest tightness, chest pain, dyspnoea, myalgia, headache, and fatigue), laboratory values, and radiologic findings on admission, treatment [proton pump inhibitors (PPIs), antivirals, antibiotics, corticosteroids, and high-flow nasal oxygen therapy], as well as complications [ARDS, acute kidney injury (AKI), and acute liver injury] and mortality status. All data were double checked independently and further verifications were done wherever necessary.

### Definition

The definition of patients with GI symptoms is that the patients had at least one of the GI symptoms of abdominal pain, acid reflux, nausea or vomiting, abdominal distension, diarrhea, tenesmus, and belching. Fever was defined as having an axillary temperature of >37.3°C. Lymphocytopenia was defined as lymphocyte count <0.8 × 109/L (26). The patients with COVID-19 were divided into four grades according to the degree of disease severity, based on the Chinese management guideline for COVID-19 (5th−7th edition) (23–25): Mild (slight clinical symptoms without CT imaging features of pneumonia); Moderate (fever and/or respiratory symptoms plus imaging features of COVID-19 pneumonia); Severe [respiratory distress (respiratory rate ≥30 breaths/min) together with the oxygen saturation ≤93% or arterial oxygen pressure (PaO_2_)/fractional inspired oxygen (FiO_2_) ratio ≤300 mmHg]; Critical [respiratory failure requiring mechanical ventilation or multiorgan failure requiring intensive care unit (ICU) admission]. Acute respiratory distress syndrome was diagnosed according to the Berlin definition (27). Acute kidney injury was identified on the basis of serum creatinine level according to the KDIGO clinical practice guideline (28). The definition of liver damage was alanine aminotransferase (ALT) >50 U/L or aspartate aminotransferase (AST) >40 U/L (29).

### Outcomes

The correlation of the GI symptoms of COVID-19 associated with mortality and other clinical features and interventions including ARDS, non-invasive ventilator treatment, tracheal intubation, tracheotomy, and continuous renal replacement therapy (CRRT) were analyzed. Other outcomes including the rate of SARS-CoV-2-related AKI, acute liver injury and the proportion of patients requiring high-flow nasal oxygen therapy, non-invasive mechanical ventilation, tracheal intubation, tracheotomy, CRRT, and ICU admission were also analyzed.

### Statistical Analyses

Given the differences in the baseline characteristics between eligible participants in the two groups, propensity-score matching was used to authenticate a cohort of patients with similar baseline characteristics. The propensity score is a conditional probability of having a particular exposure (COVID-19 patients with GI symptoms vs. without GI symptoms) given a set of baseline measured covariates. The propensity score was estimated, with COVID-19 patients with GI symptoms as the dependent variable, and age, sex, exposure history, comorbidities as covariates. Matching was performed with the use of a 1:2 matching protocol without replacement (greedy-matching algorithm), with a caliper width equal to 0.2 of the standard deviation of the logit of the propensity score. Standardized differences and *p*-values were estimated for all the baseline covariates before and after matching to assess pre-match imbalance and post-match balance. Standardized differences of <10.0% for a given covariate indicate a relatively small imbalance.

Continuous and categorical variables were presented as median (IQR) and *n* (%), respectively. We used the Mann-Whitney U-test, χ^2^-test, or Fisher's exact-test to compare differences between patients with and without GI symptoms where appropriate. Survival was estimated using the Kaplan-Meier method, and any differences in survival were evaluated with a stratified log-rank-test. To explore the GI symptoms associated with ARDS, non-invasive ventilator treatment, tracheal intubation, tracheotomy, and CRRT, univariable and multivariable COX regression models were used. A two-sided α of <0.05 was considered statistically significant. Data were analyzed with the use of the statistical packages R (The R Foundation; http://www.r-project.org; version 3.4.3 2018–02-18) and EmpowerStats (www.empowerstats.com; X&Y Solutions Inc.).

## Results

### Demographic and Epidemiological Characteristics

**Figure 1:**
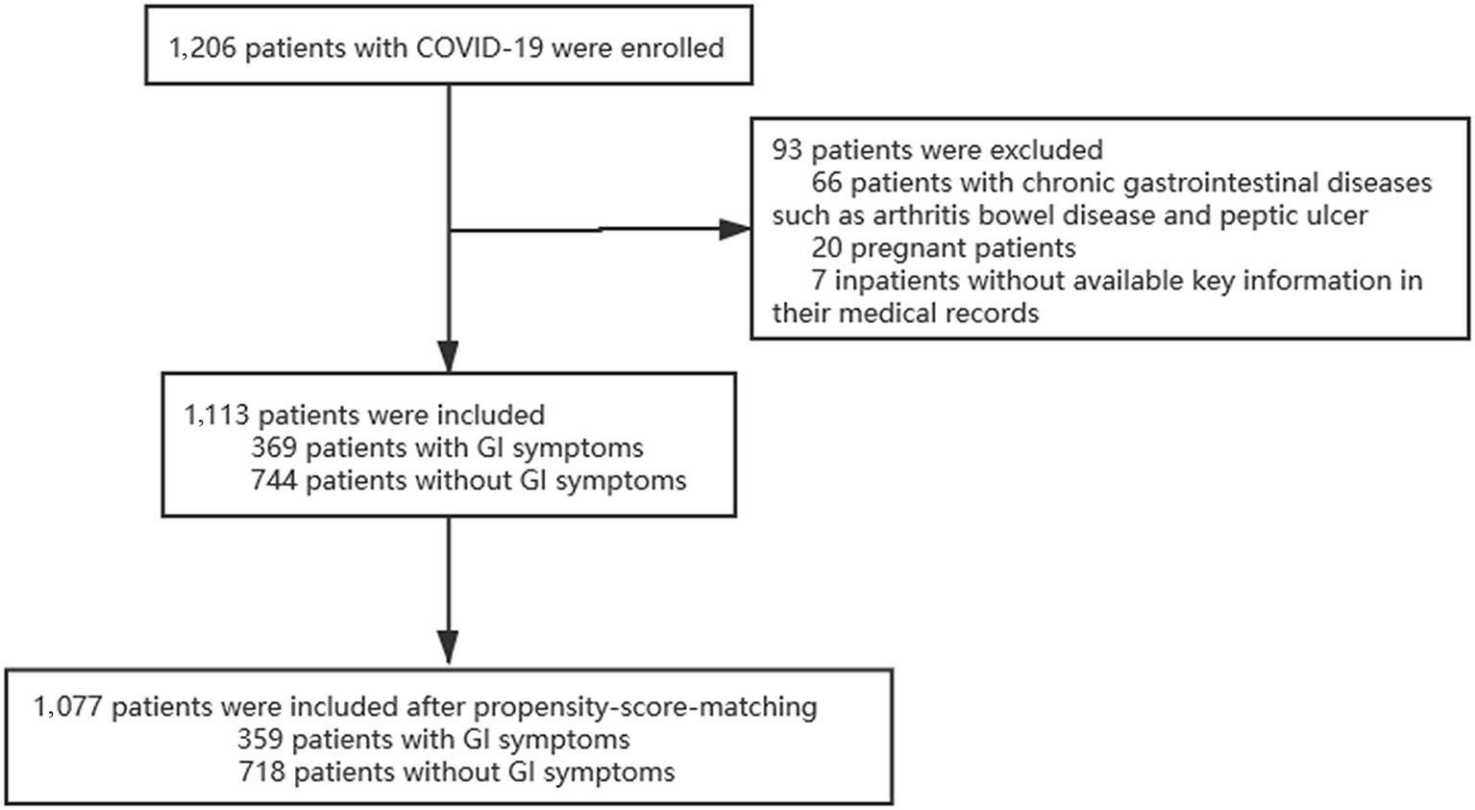
Enrollment flowchart. GI, gastrointestinal injury.

A total of 1,206 adult patients of COVID-19 were enrolled in our study from 25 January, 2020 to 31 March, 2020 in East Campus of Renmin hospital of Wuhan university; Of those, 93 were considered to be ineligible, including 66 patients with chronic gastrointestinal disease, 20 patients who were pregnant and 7 patients missing key information in their medical records. Final 1,077 patients were included in our study (Figure 1). There were differences between the two groups in several of the baseline variables before propensity score matching (PSM). After excluded 36 patients with PSM, 359 patients with GI symptoms were matched against 718 patients without GI symptoms. Their demographic data and other characteristics including comorbidities are presented in the Table 1.

**Table 1:** Baseline characteristics of patients with COVID-19 before and after propensity score matching.

**Characteristic**	**Before matching**				**After matching**			
	**All patients (*n* = 1,113)**	**Patients with GI symptoms (*n* = 369)**	**Patients without GI symptoms (*n* = 744)**	***p*-value**	**All patients (*n* = 1,077)**	**Patients with GI symptoms (*n* = 359)**	**Patients without GI symptoms (*n* = 718)**	***p*-value**
Age, year								
Median (IQR)	59.0 (47.0–68.0)	61.0 (50.0–70.0)	57.5 (46.0–67.0)	<0.001	59.0 (47.0–68.0)	60.0 (48.0–70.0)	59.0 (47.0–67.0)	0.065
Distribution, *n* (%)								
<15	0	0	0		0	0	0	
15–44	238 (21.4)	65 (17.6)	173 (23.3)		219 (20.0)	65 (18.1)	154 (21.5)	
45–64	476 (42.8)	152 (41.2)	324 (43.5)		476 (42.7)	152 (42.3)	324 (45.1)	
≥65	399 (35.8)	152 (41.2)	247 (33.2)		382 (34.3)	142 (39.6)	240 (33.4)	
Sex, *n* (%)				0.496				0.763
Male	550 (49.4)	177 (48.0)	373 (50.1)		532 (49.4)	175 (48.7)	357 (49.7)	
Female	563 (50.6)	192 (52.0)	371 (49.9)		545 (50.6)	184 (51.3)	361 (50.3)	
Exposure history, *n* (%)	153 (13.7)	49 (13.3)	104 (14.0)	0.750	152 (14.1)	48 (13.4)	104 (14.5)	0.621
Comorbidity, *n* (%)	574 (51.6)	203 (55.0)	371 (49.9)	0.106	557 (51.7)	193 (53.8)	364 (50.7)	0.343
Hypertension	368 (33.1)	133 (36.0)	235 (31.6)	0.137	355 (33.0)	124 (34.5)	231 (32.2)	0.436
Diabetes	150 (13.5)	59 (16.0)	91 (12.2)	0.084	145 (13.5)	55 (15.3)	90 (12.5)	0.207
CHD	91 (8.2)	32 (8.7)	59 (7.9)	0.671	86 (8.0)	29 (8.1)	57 (7.9)	0.937
Cerebrovascular disease	31 (2.8)	12 (3.3)	19 (2.6)	0.505	27 (2.5)	10 (2.8)	17 (2.4)	0.683
Chronic heart failure	35 (3.1)	18 (4.9)	17 (2.3)	0.020	32 (3.0)	16 (4.5)	16 (2.2)	0.042
Liver dysfunction	89 (8.0)	29 (7.9)	60 (8.1)	0.905	87 (8.1)	28 (7.8)	59 (8.2)	0.812
CKD	57 (5.1)	29 (7.9)	28 (3.8)	0.004	52 (4.8)	25 (7.0)	27 (3.8)	0.056
Chronic pulmonary disease	59 (5.3)	27 (7.3)	32 (4.3)	0.035	58 (5.4)	26 (7.2)	32 (4.5)	0.174

*Data are shown as median (IQR) or n (%). p-values were calculated using Mann-Whitney U-test, χ^2^-test, or Fisher's exact-test, as appropriate. GI, gastrointestinal; CHD, Coronary heart disease; CKD, Chronic kidney disease*.

### Clinical, Laboratory, and Radiographic Characteristics

Diarrhea (208, 57.9%), nausea or vomiting (71, 19.8%), abdominal pain (38, 10.6%) and abdominal distension (38, 10.6%) were the most frequently observed GI manifestations (Table 2). For those 359 patients with GI symptoms, 107 (29.8%) were present on initial presentation and 252 (70.2%) were present during hospitalization. Fever, cough, fatigue, chest tightness, and dyspnoea were the most common symptoms amongst all COVID-19 patients; Patients with GI symptoms had fever (287, 79.9%; *p* < 0.001), cough (168, 46.8%; <0.001), fatigue (103, 28.7%; <0.001), chest tightness (60, 16.7%; 0.019), and dyspnoea (52, 14.5%; <0.001), which were significantly higher than those in patients without GI symptoms (Table 2). Lymphocyte count in COVID-19 patients with GI symptoms was significantly lower than that in patients without GI symptoms [1.0 (0.7–1.4) vs. 1.3 (0.9–1.7), *p* < 0.001]; Lymphocytopenia occurred in 122 (34%) patients with GI symptoms. AST [27.0 (20.0–40.0) vs. 24.0 (18.0–38.0), *p* = 0.008], LDH [258.0 (200.0–355.0) vs. 227.0 (181.0–309.0)], CRP [30.8 (9.4–68.9) vs. 9.4 (2.4–54.1), *p* < 0.001] and procalcitonin [0.06 (0.04–0.14) vs. 0.05 (0.03–0.09), *p* < 0.001] were substantially higher in the COVID-19 patients with GI symptoms. Moreover, although most radiographic findings were similar between COVID-19 patients with and without GI symptoms, the rate of bilateral lung pneumonia in COVID-19 patients with GI symptoms was much higher than that in patients without GI symptoms [301 (83.8%) vs. 542 (75.5%), *p* = 0.002]. All these comparisons in the two groups are presented in the Table 3.

**Table 2:** Clinical features, disease classification of patients with COVID-19 with and without GI symptoms.

	**All patient (*n* = 1077)**	**Patients with GI symptoms (*n* = 359)**	**Patients without GI symptoms (*n* = 718)**	***p*-value**
GI symptoms				
Abdominal pain	38 (3.5)	38 (10.6)	0 (0)	
Acid reflux	12 (1.1)	12 (3.3)	0 (0)	
Nausea or vomiting	71 (6.6)	71 (19.8)	0 (0)	
Abdominal distension	38 (3.5)	38 (10.6)	0 (0)	
Diarrhea	208 (19.3)	208 (57.9)	0 (0)	
Tenesmus	9 (0.8)	9 (2.5)	0 (0)	
Belching	6 (0.6)	6 (1.7)	0 (0)	
Other symptoms				
Fever	777 (72.1)	287 (79.9)	490 (68.2)	<0.001
Cough	267 (24.8)	168 (46.8)	99 (13.8)	<0.001
Chest tightness	143 (13.3)	60 (16.7)	83 (11.6)	0.019
Chest pain	20 (1.9)	6 (1.7)	14 (1.9)	0.816
Dyspnoea	105 (9.7)	52 (14.5)	53 (7.4)	<0.001
Myalgia	61 (5.7)	31 (8.6)	30 (4.2)	0.003
Headache	28 (2.6)	12 (3.3)	16 (2.2)	0.311
Fatigue	240 (22.3)	103 (28.7)	137 (19.1)	<0.001
Time of onset of GI symptoms				
On initial presentation	107 (9.9)	107 (29.8)	0 (0)	
During hospitalization	252 (23.4)	252 (70.2)	0 (0)	
Disease classification				<0.001^*^
Mild	29 (2.7)	2 (0.5)	27 (3.8)	
Moderate	485 (45.0)	118 (32.9)	367 (51.1)	
Severe	502 (46.6)	212 (59.1)	290 (40.4)	
Critical	61 (5.7)	27 (7.5)	34 (4.7)	

*Data are median (IQR) or n (%). p-values were calculated using Mann-Whitney U-test, χ^2^-test, or Fisher's exact-test, as appropriate. GI, gastrointestinal*.

* *χ^2^-test comparing all subcategories*.

**Table 3:** Laboratory and radiographic findings of patients with COVID-19 with and without GI symptoms.

	**All patients (*n* = 1,077)**	**Patients with GI symptoms (*n* = 359)**	**Patients without GI symptoms (*n* = 718)**	***p*-value**
Laboratory findings				
White blood cell count, × 10^9^/L	5.6 (4.4–7.3)	5.5 (4.2–7.3)	5.7 (4.5–7.3)	0.091
Neutrophil count, × 10^9^/L	3.6 (2.5–5.3)	3.7 (2.5–5.6)	3.6 (2.6–5.3)	0.910
Lymphocyte count, × 10^9^/L	1.1 (0.8–1.6)	1.0 (0.7–1.4)	1.3 (0.9–1.7)	<0.001
Hemoglobin, g/L	125.0 (114.0–135.0)	125.0 (114.0–133.0)	125.0 (115.0–135.0)	0.531
Anemia	381 (35.4)	132 (36.8)	249 (34.7)	0.499
Platelet count, × 10^9^/L	214 (168.0–273.0)	207 (158.0–270.5)	219.0 (174.0–277.0)	0.006
Albumin, g/L	38.0 (34.4–41.1)	36.8 (33.7–40.0)	38.5 (34.7–41.4)	<0.001
ALT, U/L	25.0 (17.0–42.0)	26.0 (17.0–41.0)	24.0 (16.0–42.0)	0.607
AST, U/L	25.0 (19.0–38.0)	27.0 (20.0–40.0)	24.0 (18.0–38.0)	0.008
Urea, mmol/L	4.7 (3.7–6.2)	4.8 (3.7–6.5)	4.7 (3.7–6.1)	0.332
Creatinine, μmol/L	59.0 (49.0–71.0)	59.0 (49.0–73.0)	59.0 (50.0–70.0)	0.639
LDH, U/L	237.0 (188.0–325.0)	258.0 (200.0–355.0)	227.0 (181.0–309.0)	<0.001
PT, s	11.9 (11.2–12.6)	12.0 (11.3–12.7)	11.8 (11.2–12.5)	0.008
APTT, s	27.5 (25.6–29.9)	27.8 (25.7–30.8)	27.3 (29.4–25.5)	0.009
CRP, mg/L	16.8 (3.0–60.5)	30.8 (9.4–68.9)	9.4 (2.4–54.1)	<0.001
D-dimer, μg/mL	0.7 (0.4–1.9)	0.9 (0.4–2.5)	0.6 (0.3–1.6)	<0.001
Procalcitonin, ng/mL	0.05 (0.03–0.10)	0.06 (0.04–0.14)	0.05 (0.03–0.09)	<0.001
Glu, mmol/L	5.5 (4.8–6.9)	5.6 (4.9–7.1)	5.3 (4.8–6.7)	0.007
Na, mmol/L	141.0 (139.0–145.0)	141.0 (138.0–144.0)	142.0 (139.0–145.0)	0.009
K, mmol/L	4.0 (3.7–4.3)	4.0 (3.6–4.4)	4.0 (3.7–4.3)	0.376
Ca, mmol/L	2.1 (2.0–2.2)	2.1 (2.0–2.2)	2.2 (2.1–2.3)	<0.001
Radiologic findings				
Bilateral	843 (78.3)	301 (83.8)	542 (75.5)	0.002
Ground-glass opacity	656 (60.9)	225 (62.7)	431 (60.0)	0.401
Patchy shadows	612 (56.8)	165 (46.0)	447 (62.3)	<0.001
Diffuse interstitial infiltrations	14 (1.3)	8 (2.2)	6 (0.8)	0.083

*Data are median (IQR) or n (%). p-values were calculated using Mann-Whitney U-test, χ^2^-test, or Fisher's exact-test, as appropriate. GI, gastrointestinal; ALT, alanine aminotransferase; AST, aspartate aminotransferase; LDH, lactic dehydrogenase; PT, prothrombin time; APTT, activated partial thromboplastin time; CRP, C-reactive protein*.

### Treatment, Complications, and Clinical Outcomes

The number of patients receiving antivirals [342 (95.3%) vs. 648 (90.3%), *p* = 0.004], antibiotics [286 (79.7%) vs. 479 (66.7%), *p* < 0.001], and corticosteroids [148 (41.2) vs. 244 (34.0), *p* = 0.020] were significantly different between the COVID-19 patients with and without GI symptoms (Table 4). 298 (83.0%) COVID-19 patients with GI symptoms were treated with high-flow nasal oxygen therapy, 54 (15%) with non-invasive mechanical ventilation, 20 (5.6%) with tracheal intubation ventilation, 7 (1.9%) with CRRT, which were higher than those in patients without GI symptoms, respectively (Table 4). Acute respiratory distress syndrome was the most frequently observed complication, in addition to AKI and acute liver injury. The rate of ARDS in patients with GI symptoms was higher than that in patients without GI symptoms [72 (20.1%) vs. 61 (8.5), *p* < 0.001]. As of March 31, 785 (72.9%) patients with COVID-19 have been discharged from hospital, and 207 (19.2%) patients remained in hospital.

**Table 4:** Treatment, complications and clinical outcomes of patients with COVID-19 with or without GI symptoms.

	**All patients (*n* = 1,077)**	**Patients with GI symptoms (*n* = 359)**	**Patients without GI symptoms (*n* = 718)**	***p*-value**
Treatments				
PPIs	460 (42.7)	205 (57.1)	255 (35.5)	<0.001
Antivirals	990 (91.9)	342 (95.3)	648 (90.3)	0.004
Antibiotics	765 (71.0)	286 (79.7)	479 (66.7)	<0.001
Corticosteroids	392 (36.4)	148 (41.2)	244 (34.0)	0.020
High-flow nasal oxygen therapy	849 (78.8)	298 (83.0)	551 (76.7)	0.018
Non-invasive mechanical ventilation	99 (9.2)	54 (15.0)	45 (6.3)	<0.001
Tracheal intubation	33 (3.1)	20 (5.6)	13 (1.8)	<0.001
Tracheotomy	8 (0.7)	4 (1.1)	4 (0.6)	0.452
CRRT	9 (0.8)	7 (1.9)	2 (0.3)	0.008
ICU admission	52 (4.8)	22 (6.1)	30 (4.2)	0.159
ICU length of stay, days	14.0 (8.0–24.0)	15.5 (8.0–24.8)	14.0 (8.2–17.2)	0.498
Complications				
ARDS	133 (12.3)	72 (20.1)	61 (8.5)	<0.001
AKI	13 (1.5)	5 (1.4)	8 (1.1)	0.693
Acute liver injury	16 (1.5)	6 (1.7)	10 (1.4)	0.791
Hospital length of stay, days	16.0 (9.0–32.0)	24.8 (12.0–36.0)	14.0 (8.0–28.0)	<0.001
Clinical outcomes				
Discharge from hospital	785 (72.9)	254 (70.8)	531 (74.0)	0.265
Death	85 (7.9)	34 (9.5)	51 (7.1)	0.174
Staying in hospital	207 (19.2)	71 (19.7)	136 (19.9)	

*Data are median (IQR) or n (%). p-values were calculated using Mann-Whitney U-test, χ^2^-test, or Fisher's exact-test, as appropriate. GI, gastrointestinal; PPIs, proton pump inhibitors; CRRT, continuous renal replacement therapy; ARDS, acute respiratory distress syndrome; AKI, acute kidney injury; ICU, intensive care unit*.

### Correlation of Measures

**Figure 2:**
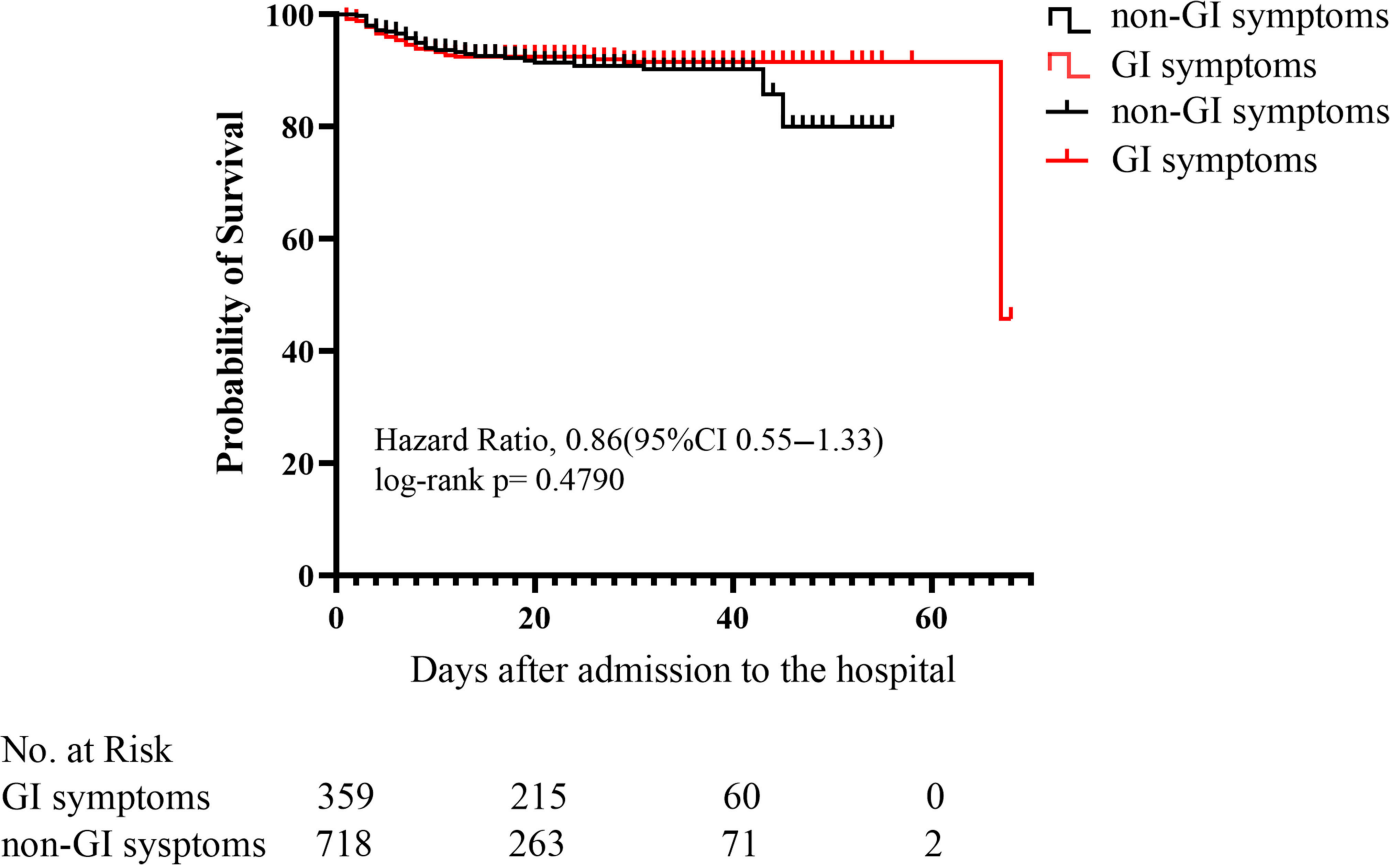
Kaplan-Meier (KM) survival curve for COVID-19 patients with or without GI (gastrointestinal) symptoms. *p*-values were for differences by log-rank-test.

Kaplan-Meier curves showed that there was no significant difference (*p* = 0.479) in mortality between COVID-19 patients with and without GI symptoms (Figure 2). The univariate regression analysis (Table 5) showed that the patients with GI symptoms was significantly associated with developing ARDS (HR 2.7, 95%CI 1.9–3.9, *p* < 0.001), requiring non-invasive mechanical ventilation (HR 2.6, 95%CI 1.7–4.0, *p* < 0.001), tracheal intubation (HR 3.2, 95%CI 1.6–6.5, *p* < 0.001), and CRRT (HR 7.1, 95%CI 1.5–34.4, *P* = 0.015). In the multivariate analysis, after the adjusting with lymphocyte, PLT, Albumin, Urea, Creatinine, LDH, PT, APTT, D-dimer, CRP, and Procalcitonin, patients with GI symptoms independently associated with non-invasive mechanical ventilation (HR 3.1, 95%CI 1.8–5.4, *p* < 0.001), tracheal intubation (HR 2.4, 95%CI 1.1–5.5, *p* = 0.037) and ARDS (HR 2.8, 95%CI 1.7–4.6, *p* < 0.001). There was no association between patients with GI symptoms and the requirement for CRRT (HR 5.1, 95%CI 0.5–53.0, *P* = 0.175). After adjusted with variables in Adjust I model and antiviral treatment, antibiotics and corticosteroids, the presence of GI symptoms remained an independent predictor for ARDS (HR 2.9, 95%CI 1.8–5.0, *p* < 0.001), non-invasive mechanical ventilation (HR 3.3, 95%CI 1.9–5.7, *p* < 0.001), and tracheal intubation (HR 2.5, 95%CI 1.1–6.0, *p* = 0.035) in COVID-19 patients.

**Table 5:** Univariate and multivariate analysis for non-invasive mechanical ventilation, tracheal intubation, CRRT, and ARDS in COVID-19 patients with GI symptoms.

	**Hazare ratio**	**95%CI**	***p*-value**
ARDS			
Unadjusted	2.7	1.9–3.9	<0.001
Adjusted I	2.8	1.7–4.6	<0.001
Adjusted II	2.9	1.8–5.0	<0.001
Non-invasive mechanical ventilation			
Unadjusted	2.6	1.7–4.0	<0.001
Adjusted I	3.1	1.8–5.4	<0.001
Adjusted II	3.3	1.9–5.7	<0.001
Tracheal intubation			
Unadjusted	3.2	1.6–6.5	0.001
Adjusted I	2.4	1.1–5.5	0.037
Adjusted II	2.5	1.1–6.0	0.035
CRRT			
Unadjusted	7.1	1.5–34.4	0.015
Adjusted I	5.1	0.5–53.0	0.175
Adjusted II	6.1	0.5–71.3	0.149

*Adjust I model adjusting for Lymphocyte, PLT, Albumin, Urea, Creatinine, LDH, PT, APTT, D-dimer, CRP, and Procalcitonin; Adjust II model adjusting by variables in Adjust I model plus antiviral treatment, antibiotics, and corticosteroids. CRRT, continuous renal replacement therapy; ARDS, acute respiratory distress syndrome*.

## Discussion

This retrospective cohort study of patients with COVID-19 showed that gastrointestinal (GI) symptoms were associated with a higher risk of ARDS, non-invasive mechanical ventilation, and tracheal intubation. The risk of death was similar amongst COVID-19 patients with or without GI symptoms. Many studies have confirmed that GI symptoms in COVID-19 patients are associated with the disease prognosis. Hajifathalian et al. (30) reported a lower mortality rate in patients with GI symptoms compared to those without any GI symptoms. Another study from Spain involving 2,226 patients with COVID-19 came to similar conclusions (31). In contrast, many studies have shown that GI symptoms are associated with poor prognosis. A meta-analysis reported that patients with GI symptoms had a higher rate of severe or critical COVID-19 infection compared to patients without any GI symptoms (32). To our knowledge, this is the first study examining the relationship between GI symptoms and prognosis in patients with COVID-19 with a relatively large sample size. In previously published studies of COVID-19 patients with GI symptoms, the patient numbers were too small to conclude the characteristics and mortality of these patients with SARS-CoV-2 pneumonia (29, 33, 34).

Although COVID-19 is characterized by respiratory tract manifestations, GI symptoms are not uncommon. In some cases, GI symptoms, particularly diarrhea, can be the initial presentation of COVID-19 in patients who may later (or never) present with respiratory symptoms (35). Moreover, another research from Wuhan showed that patients with GI symptoms risked not being promptly recognized, leading to a delayed diagnosis of COVID-19 (12). These patients were diagnosed as COVID-19 positive with SARS-CoV-2 nucleic acid in stool or rectal swabs (36). Among the total of 1,113 COVID-19 patients enrolled, the rate of patients with GI symptoms was 33.2%, which was higher than the data reported previously (3, 29). The reason of this discrepancy is unknown but may be related to the main task of East Campus of Renmin Hospital of Wuhan University in undertaking the treatment of critical COVID-19 patients.

In our study, the clinical characteristics of COVID-19 patients with GI symptoms were a significantly higher rate of fever, cough, chest tightness, dyspnoea, myalgia and fatigue, and had increased complication of ARDS and a higher tendency toward higher disease severity (rate of severe/critical type and mechanical ventilation) compared with COVID-19 patients without GI symptoms, which is consistent with a study reported previously (29). This may be related to bacterial translocation and electrolyte disturbances, as evidenced by significantly increased CRP and procalcitonin levels, decreased lymphocyte count and serum sodium levels. In addition, although the incidence of AKI and acute liver injury was similar between the COVID-19 patients with or without GI symptoms, the AST level and creatinine above 133 μmol/L in the COVID-19 patients with GI symptoms were higher than those without GI symptoms. These results highlighted the need to closely monitor liver and kidney functions during the course of the disease.

The functional host cell “receptor” for SARS-CoV-2 is angiotensin converting enzyme 2 (ACE2) (37, 38). The spike glycoprotein (S protein) on the virion surface mediates receptor recognition and membrane fusion, thus exploiting ACE2 for host infection (39). ACE2 receptors are not only distributed in bronchial transient secretory cells (40) but also in various tissues and organs, such as kidneys, small intestine, (41) and testis (42). In the intestines, ACE2 is primarily distributed on the luminal surface of differentiated small intestinal epithelial cells, and is identified as a key regulator of dietary amino acid homeostasis, innate immunity, gut microbial ecology, and transmissible susceptibility to colitis (43). These may mediate the invasion of the virus, activation and amplification of gastrointestinal inflammation (44) and lead to GI symptoms in patients with COVID-19.

The gastrointestinal tract represents a large microbial ecosystem, housing several trillion microbiota. Under normal circumstances, the intestinal microbiota plays a critical role in the maturation of the host immune response (45), influences the regulation of intestinal endocrine functions (46) and maintains the homeostasis of gastrointestinal tract. An increase in gut permeability, bacterial translocation and local responses can be found in patients with critical illness of various causes (47). For example, in intestinal ischemia-reperfusion injury, it has been demonstrated that the reperfused gut can become a source of pro-inflammatory mediators (48) which can be delivered to remote organs and amplify the early systemic inflammatory response (22). Consistent with the results of previous animal studies (22), the presence of GI symptoms is associated with a higher rate of ARDS, non-invasive mechanical ventilation and tracheal intubation in patients with COVID-19. However, our study showed that GI symptoms did not appear to affect the mortality rate among COVID-19 patients but the sample size under power to detect any statistical significances of mortality can not be excluded. Furthermore, at the point of data analysis, some patients were still in the hospital and their long term outcomes are unknown whilst the retrospective nature and a single-center data of our study would call more studies into this during the disease pandemic.

In conclusion, this work is one of the largest cohort of COVID-19 patients with GI symptoms. COVID-19 patients with GI symptoms, as compared with absence of GI symptoms, were associated with high risks of ARDS, non-invasive mechanical ventilation, and tracheal intubation. Therefore, we should pay greater attention to COVID-19 patients with GI and other non-classical symptoms for better care of our patients and remain vigilant in the protection of healthcare providers.

## Data Availability Statement

The raw data supporting the conclusions of this article will be made available by the authors, without undue reservation.

## Ethics Statement

The studies involving human participants were reviewed and approved by Institutional Review Board at Renmin hospital of Wuhan University (No. WDRY2020-K111, March 12, 2020). Written informed consent for participation was not required for this study in accordance with the national legislation and the institutional requirements.

## Author Contributions

LG, Q-tM, and DM designing research studies, reviewed, and edited the manuscript. YL and J-xL acquiring data. RC, Y-lY, and WL analyzing data and writing the paper. Illustrations and proofreading were performed by YL, FC, and QZ. All authors read and approved the manuscript.

## Conflict of Interest

The authors declare that the research was conducted in the absence of any commercial or financial relationships that could be construed as a potential conflict of interest.
